# Synergistic Anti-*Helicobacter pylori* Effects of *Takifugu obscurus* Skin Peptides and *Lactobacillus plantarum*: A Potential Gastric Health Dietary Supplement

**DOI:** 10.3390/foods14030406

**Published:** 2025-01-26

**Authors:** Lei Gu, Yiying Tang, Jieshuai Zhang, Ningping Tao, Xichang Wang, Liping Wang, Changhua Xu

**Affiliations:** 1 College of Food Science & Technology, Shanghai Ocean University, Shanghai 201306, China; leigu1023@163.com (L.G.); tangyiying1998@outlook.com (Y.T.); zhangjs277@163.com (J.Z.); nptao@shou.edu.cn (N.T.); xcwang@shou.edu.cn (X.W.); 2 Shanghai Engineering Research Center of Aquatic-Product Processing & Preservation, Shanghai 201306, China; 3 Laboratory of Quality and Safety Risk Assessment for Aquatic Products on Storage and Preservation (Shanghai), Ministry of Agriculture, Shanghai 201306, China; 4 National R&D Branch Center for Freshwater Aquatic Products Processing Technology (Shanghai), Shanghai 201306, China

**Keywords:** bioactive peptides, enzymolysis, food-derived, *Lactobacillus plantarum*, *Helicobacter pylori*, antibacterial activity, dietary supplement

## Abstract

*Helicobacter pylori* (*H. pylori*) infection is a widespread gastric infectious disease, posing significant challenges due to the increasing prevalence of antibiotic resistance. This study aimed to evaluate the synergistic antibacterial activity of *Takifugu obscurus* skin peptides (TSPs) and the cell-free supernatant of *Lactobacillus plantarum WUH3* (LCFS) in developing a potential green and efficient dietary supplement therapy. Using enzymatic hydrolysis and ultrafiltration techniques, the most bioactive peptide fraction, TSPb (1–3 kDa), was identified. The effects of TSPb and LCFS—both individually and in combination—on *H. pylori* biofilm function, membrane morphology, and internal structure were systematically analyzed using urease activity, N-phenyl naphthylamine (NPN) uptake, nucleic acid leakage, scanning electron microscopy (SEM), and infrared (IR) spectroscopy. The results showed that both LCFS and TSPb significantly inhibited *H. pylori* urease activity, with inhibition rates of 53.60% and 54.21% at 24 h, respectively, and the highest inhibition rate of 74.64% was observed with their combined treatment. SEM, NPN fluorescence, and nucleic acid leakage analyses revealed distinct mechanisms of action for each treatment. LCFS treatment caused membrane surface loosening and morphological deformation, while TSPb induced the formation of localized membrane pores. IR spectroscopy further confirmed that the combined treatment led to a more severe disruption of the lipid and protein structure within the bacterium. Overall, compared to individual treatments, the combination of TSPb and LCFS exhibited enhanced intracellular penetration and a more significant effect on bacterial viability. This study successfully identified TSPb as a highly bioactive peptide and elucidated its potential synergistic antibacterial mechanism with LCFS. These findings provide scientific evidence for the development of functional antimicrobial foods and gastric health dietary supplements, offering a promising strategy for the prevention and management of *H. pylori* infections.

## 1. Introduction

*H. pylori* is a microaerophilic Gram-negative bacterium that colonizes the human stomach effectively due to its spiral cell shape and single-ended sheathed flagellum [1]. Prolonged adherence of *H. pylori* to the human gastric mucosa can lead to various diseases, such as inflammation, gastric ulcers, gastric cancer, and gastric mucosa-associated lymphoma [2,3]. According to the World Health Organization, approximately 50% of the global population is infected with *H. pylori* [4]. Currently, triple therapy consisting of a proton pump inhibitor or a colloidal bismuth agent plus two antibiotics is commonly used in clinical practice, including metronidazole, azithromycin, clarithromycin, amoxicillin (AMX), tetracycline, furazolidone, and levofloxacin [5]. However, with the widespread use of antibiotics, the antibiotic resistance rate of *H. pylori* strains is rapidly increasing, the eradication rate of *H. pylori* is decreasing, and recurrence of peptic ulcers has become a common clinical problem, making the development of new treatments more urgent [6].

Probiotics have gained popularity in recent years because of their ability to improve the eradication rate of some harmful bacteria with minimal side effects. Their secretion of antibacterial substances can effectively hinder the adhesion and colonization of harmful bacteria in the gastric mucosa, regulate the immune response of host organisms, promote the secretion of anti-inflammatory cytokines, and improve both the inflammatory response and tissue activity [7,8]. Probiotics can produce a wide range of antimicrobial compounds, such as bacteriocins, ethanol, organic acids, diacetyl, acetaldehyde, hydrogen peroxide (H_2_O_2_), and peptides [9]. These compounds increase the permeability of target bacterial cell membranes and polarize the cell membrane, and the H_2_O_2_ produced by probiotics can oxidize thiol groups, leading to the denaturation of membrane lipids and ultimately resulting in cell death [10]. *Lactobacillus plantarum*, as a member of probiotics, can not only reduce the infection rate and invasiveness of *H. pylori* and improve gastrointestinal tract function but also enhance the function of the mucosal barrier and reduce the risk of ulceration and carcinoma after *H. pylori* infection [11,12]. Previous studies have confirmed that *Lactobacillus plantarum WUH3* produces the class IIb bacteriocin Plantaricin E, along with potentially various other bioactive substances (such as hydrogen peroxide, lactic acid, and phospholipase), demonstrating its potential as a natural biotherapeutic agent against gastrointestinal pathogens like *H. pylori* [13].

Antimicrobial peptides (AMPs) are a new class of antimicrobial agents with broad-spectrum activity, unique spatial structures, and distinct mechanisms of action. They are not subject to issues of drug resistance and mutation [14]. Although the exact mechanism of action of AMPs has not been fully elucidated, current research suggests that it primarily involves a disruption of the membrane structure or non-membrane structures [15]. In the membrane disruption mode, positively charged AMPs can adsorb to the biofilm through electrostatic interactions, forming transmembrane channels and disrupting the biofilm to cause cell death. Additionally, for some bacteria that can survive membrane disruption, AMPs can penetrate the cell interior and completely kill the bacteria by inhibiting the synthesis of nucleic acids and proteins. The non-membrane disruption mode is less common and involves rapid passage through the biofilm without causing rupture, instead targeting intracellular components to interfere with critical physiological processes. AMPs bind to intracellular target molecules and significantly disrupt intracellular physiological processes, such as nucleic acid metabolism, protein synthesis, and cellular signal transduction, thereby inactivating the bacteria [16,17].

Although AMPs demonstrate significant potential antimicrobial activity, particularly in combating multidrug-resistant bacteria such as *H. pylori*, they still face several challenges in current applications [18]. One major issue with AMPs is their potential cytotoxicity, which is often concentration-dependent. For instance, certain AMPs, such as melittin and alamethicin, can cause hemolysis and tissue damage at higher concentrations [19]. However, studies have shown that food-derived antimicrobial peptides, such as those from dairy products, fish, and plants, exhibit low toxicity and good biocompatibility, making them promising candidates for both pharmaceutical and food applications. For example, antimicrobial peptides extracted from soybeans, dairy products, and wheat have shown low toxicity in vitro and in animal experiments, typically without affecting normal cellular functions [20]. *Takifugu obscurus* skin is rich in collagen, but as a processing byproduct, it is typically discarded as waste. However, it has been shown that fish skin peptides are used for a variety of physiological functions; for example, peptides extracted from the skin of *Sphyrna mokarran* improve the effect of oxidative stress on the gastric mucosa of ulcerated rats, peptides extracted from *Tilapia* skin have anti-inflammatory and wound healing-promoting effects, and skin peptides from *Takifugu obscurus* can effectively inhibit the growth of ice crystals, etc. [21,22,23]. However, there are few reports on the application of *Takifugu obscurus* skin peptides in antimicrobial activity.

Currently, only limited research has focused on the combined antimicrobial effects of AMPs and probiotic supernatants. AMPs face several stability issues, as they are often susceptible to enzymatic degradation and changes in pH—particularly in the gastrointestinal tract—which can reduce their antimicrobial efficacy. Factors such as gastric acid and digestive enzymes can negatively impact the stability of AMPs. However, the organic acids and metabolic products in probiotic supernatants may enhance the stability of AMPs in the gastrointestinal tract by forming a protective barrier, preventing degradation by gastric acid and digestive enzymes [20]. AMPs provide direct antimicrobial activity, rapidly inhibiting the growth of pathogens such as *H. pylori*, while probiotic supernatants exert supportive effects through mechanisms such as immune modulation, inhibition of pathogen colonization, and alteration of the microbial environment. This synergistic approach boosts overall antimicrobial efficacy, reduces the likelihood of pathogen resistance mutations, and lowers the risk of resistance development [24].

This study aims to evaluate the inhibitory effects and potential mechanisms of the combination of *Takifugu obscurus* skin-extracted peptides and the supernatant of *Lactobacillus plantarum* WUH3 on *H. pylori* by assessing urease activity, biofilm integrity, and nucleic acid leakage levels, as well as through scanning electron microscopy (SEM) and infrared spectroscopy. This study explores the potential of combining antimicrobial peptides and probiotics as an alternative strategy for the prevention and treatment of *H. pylori* infections, providing new approaches for the development of functional foods and dietary supplements targeting gastric health.

## 2. Materials and Methods

### 2.1. Materials and Reagents

Takifugu obscurus skin was obtained from Zhejiang Xingye Group Co. (Zhoushan, China) The protease complex was purchased from Novozymes (China) Biotechnology Co. (Beijiing, China) The *H. pylori* liquid medium, MRS medium, and defatted fibrous sheep blood were purchased from Shanghai Gao Xin Chemical Glass Co. (Shanghai, China) Colombian blood agar plates were purchased from Shanghai Doshen Biotechnology Co. (Shanghai, China) AMX and the 1-N phenyl naphthylamine (NPN) fluorescent agent were purchased from Shanghai Maclean’s Reagent Co. (Shanghai, China) The UA assay kit was purchased from Beijing Box Biotechnology Co. (Beijiing, China). AnaeroPack microaerobic gas production bags and AnaeroPouch stand-up culture bags were purchased from Shanghai Jing Lai Trading Co. (Shanghai, China).

### 2.2. Preparation and Isolation of TSP

The Takifugu obscurus skin was cut into small pieces (approximately 1 cm × 1 cm), packed into 100 g per bag, and stored at −40 °C for later use. After being thoroughly cleaned and drained, 100 g of fish skin was placed in a 500 mL Erlenmeyer flask, and extracted at a ratio of fish skin/water = 1:3 (*w*/*v*) in a water bath at 90 °C for 3 h. It was then digested by 1.5 AU/g complex protease (obtained by fermentation with Bacillus amyloliquefaciens and Bacillus licheniformis.) in a shaking water bath at pH 7.0, with an enzyme–substrate ratio of 1:50 (*w*/*w*), a temperature of 55 °C, and 120 rpm for 2 h. The reaction was terminated by being held at 95 °C for 15 min. The cooled sample was filtered by centrifugation and decolorized by activated carbon to obtain the peptide hydrolysate TSP. The hydrolysate TSP was passed through 10 kDa, 3 kDa, and 1 kDa ultrafiltration membranes to collect the hydrolysates with molecular weights (MWs) <1 kDa (TSPa), 1–3 kDa (TSPb), 3–10 kDa (TSPc), and >10 kDa (TSPd), respectively. TSP with different MWs was obtained after being dried in a Vacuum Freeze Dryer (FD-1D-80, Beijing Boyikang Experimental Instrument Co., Beijiing, China) for 48 h.

### 2.3. Bacterial Culture and Experimental Grouping

H. pylori was provided by Shanghai Yiyan Biotechnology Co., Ltd., (Shanghai, China), strain number ATCC 43504, and stored frozen in glycerol at −80 °C. The AnaeroPack microaerobic pouch and AnaeroPouch stand-up pouch were used to provide a microaerobic environment, and *H. pylori* liquid medium supplemented with defatted fibrous sheep blood was incubated at 37 °C in an incubator.

*Lactobacillus plantarum* was provided by the Key Laboratory of Microbiology, College of Food, Shanghai Ocean University, with strain number WUH3. It was also stored frozen in glycerol at −80 °C. The LCFS was obtained by filtering the supernatant through a sterile 0.22 µm filter membrane after 24 h of incubation in a sterile culture tube at 37 °C in a shaker incubator using MRS liquid medium and centrifuging at 8000 rpm for 15 min at 4 °C [25]. The TSPb and LCFS were mixed to produce an LCFS-TSPb mixture at 10:1.

*H. pylori* was divided into a control group, positive drug group (AMX, 0.125 mg/mL), TSP group, LCFS group, and LCFS+TSPb group. The different additions were using *H. pylori* liquid medium, and TSP-1, TSP-2, and TSP-3 represented TSP concentrations of 1 mg/mL, 2 mg/mL, and 3 mg/mL, respectively.

### 2.4. Analysis of the Amino Acid Distribution

An appropriate amount of TSP sample was weighed into a hydrolysis tube, acidified with 10 mL of hydrochloric acid (6 mol/L), and then sealed under vacuum. After high-temperature hydrolysis, filtration, and vacuum drying, the residues were fully dissolved and filtered using a sodium citrate solution with a pH of 2.2. The amino acid composition was determined using an ultra-high-speed amino acid auto-analyzer (LA-8080, Hitachi, Tokyo, Japan).

### 2.5. SEM Morphological Characterization

Take 20 μL of the separated and treated bacterial sample, drop it onto a coverslip, and allow it to air dry. Coat the sample with gold by sputtering and then perform the analysis using the instrument (sigma HD, Zeiss, Oberkochen, German).

### 2.6. H. pylori Inhibition Assay

The agar well diffusion method was used to evaluate the inhibitory effect of TSP on *H. pylori* [26]. A total of 100 μL of *H. pylori* in the logarithmic growth phase was evenly coated onto blood agar plates. Filter paper sheets were then evenly distributed onto the plates, and 150 μL of drugs (AMX and TSP) prepared in *H. pylori* liquid medium was added dropwise onto the filter paper sheets. The plates were incubated at 37 °C under microaerobic conditions for 48 h, and the diameter of the inhibition zone was measured.

### 2.7. H. pylori UA Assay

The assay was performed using a UA assay kit. Briefly, 100 ul of *H. pylori* in the logarithmic growth stage was added to drug-containing medium (AMX, TSP, LCFS, and LCFS-TSPb). The absorbance values at 630 nm were measured after 24 and 48 h of incubation at 37 °C under microaerobic conditions, according to the kit’s instructions.

### 2.8. Nucleic Acid Leakage Level

Nucleic acid leakage from the bacterial cells is reflected by measuring the OD at 260 nm [27]. After 100 uL of *H. pylori* in the logarithmic growth stage was added to drug-containing medium (AMX, LCFS, and LCFS-TSPb) and incubated at 37 °C for 24 and 48 h under microaerobic conditions, 1 mL of the *H. pylori* bacterial solution was taken and centrifuged at 8000 rpm for 5 min, and the supernatant and bacterial body were collected separately. The absorbance at 260 nm was measured by adding 20 μL of the supernatant to 180 μL of distilled water as a blank control.

### 2.9. NPN Absorption Assay

Changes in bacterial biofilm permeability were detected by the NPN absorption assay [28,29]. Bacteria obtained by previous centrifugation were washed three times with PBS buffer and resuspended to an OD600 = 0.5. A total of 200 μL of the bacterial suspension was mixed with 2 μL of the NPN solution, and the fluorescence intensity was quantified at excitation/emission wavelengths of 350/420 nm.

### 2.10. H. pylori IR Spectra Data Acquisition

The *H. pylori* bacterial precipitate was collected after a 48 h incubation with the drug. Subsequently, it underwent a series of washing steps, including one wash with 0.9% physiological saline and two washes with ultrapure water. The washed precipitate was then resuspended through centrifugation. For spectral acquisition, 10 μL of the bacterial solution was carefully pipetted onto the center of a calcium fluoride (CaF2) window slice measuring 12 mm × 1 mm. The sample spots were dried until visible bacterial spots appeared. Prior to analysis, atmospheric noise was removed, and baseline correction was applied to ensure accurate results.

### 2.11. Statistical Analysis

The mean ± standard deviation (SD) were used to express all data. One-way analysis of variance (ANOVA) and graphs were conducted using GraphPad Prism version 9.0 software, with error bars representing the SDs based on the means of at least three replicates. Statistically significant results were indicated by * *p* < 0.05, ** *p* < 0.01, and *** *p* < 0.001. Analyses of IR spectral and second derivative IR (SD-IR) spectral data were performed using Spectrum’s software PerkinElmer (Version 10.6.0, PerkinElmer, Inc., Springfield, IL, USA). MATLAB software 2019b (9.7.0.1190202) (The MathWorks, Natick, MA, USA) was used for homebrew scripting of the dynamic spectra of *H. pylori* after different treatments in order to obtain two-dimensional correlation infrared spectra (2DCOS-IR) based on drug perturbations.

## 3. Results

### 3.1. Anti-H. pylori Activity of TSP

The agar diffusion method was used to measure the diameter of the inhibition zones of *H. pylori* treated with different concentrations of the crude protein extract from *Takifugu obscurus* skin (TSP). As shown in Table 1, the diameter of the inhibition zones in the TSP-treated groups was positively correlated with the peptide concentration. Notably, the antimicrobial activity of the TSP-3 group (0.94 ± 0.05 cm) was 53.41% of the positive control AMX group (1.76 ± 0.25 cm) (Table 1), indicating the potential of *Takifugu obscurus* skin as a source of natural antimicrobial proteins against *H. pylori*. To further investigate the active components of TSP, the extract was fractionated into four fractions based on molecular weight using ultrafiltration.

### 3.2. UA Inhibition Rate of H. pylori

**Figure 1:**
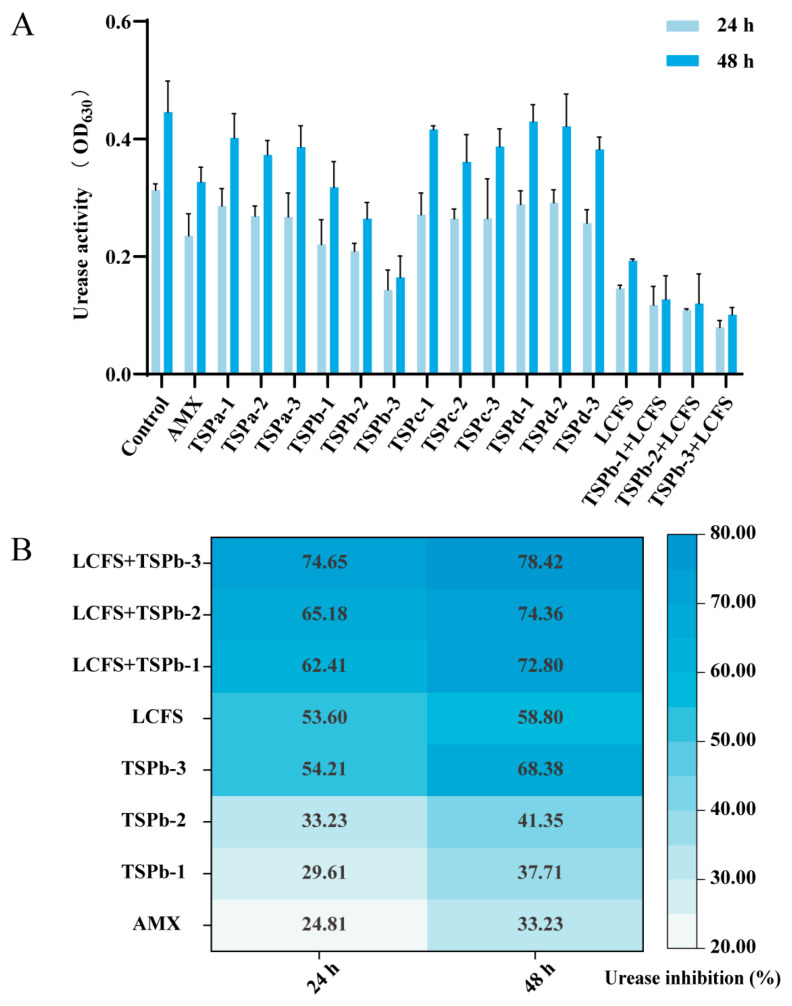
(**A**) UA of *H. pylori* with different treatments: positive drug—amoxicillin (AMX), TSPa (<1 kDa), TSPb (1–3 kDa), TSPc (3–10 kDa) and TSPd (>10 kDa) (obtained by ultrafiltration from TSP), LCFS—extract from *Lactobacillus plantarum WUH3,* and TSPb+LCFS (combination) (*n* = 3). (**B**) UA inhibition of *H. pylori* after treatment with LCFS and TSPb alone or in combination (*n* = 3).

An investigation of the absorbance values at 630 nm was essential to indicate UA levels and assess the effect of TSP on the survival environment of *H. pylori*. The absorbance of the *H. pylori* control group increased from 0.31 ± 0.01 (24 h) to 0.45 ± 0.05 (48 h) with the prolongation of the incubation time, which was higher than that of the drug-treated group. After 48 h, the absorbance of TSPb-3 group (0.16 ± 0.03) was 30.68% of the control group, which was more efficient in disrupting the living conditions of *H. pylori* than other MW peptides, making it suitable for more in-depth studies (Figure 1A).

As shown in Figure 1B, it was observed that the inhibition rate of urease activity displayed a time- and concentration-dependent relationship with TSPb within a 48 h period of drug treatment. Notably, the TSPb-3 group exhibited a remarkable inhibition rate of urease activity, reaching 68.38%. This value was twice as high as that of the AMX group, which recorded an inhibition rate of 33.23%. Furthermore, the combination group, consisting of LCFS and TSPb, demonstrated even higher inhibitory effects compared to the individual effects of LCFS and TSPb alone. This suggests a possible synergistic effect of combination therapies.

### 3.3. Characterization of TSPb

**Figure 2:**
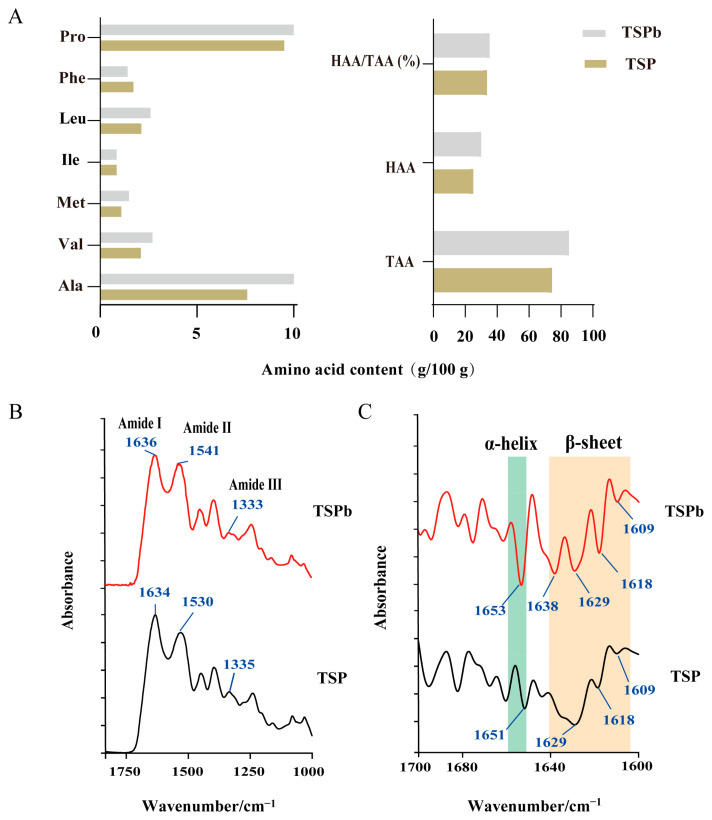
Amino acid composition and IR profiles of TSP and TSPb. (**A**) Hydrophobic amino acid (HAA) content and its percentage among the total amino acids (TAAs) (*n* = 3); (**B**) IR spectra of TSP and TSPb from 1700 to 1000 cm^−1^; (**C**) SD-IR spectra of TSP and TSPb from 1700 to 1600 cm^−1^ (*n* = 3).

A large amount of hydrophobic amino acids is beneficial for bacterial cell self-aggregation, effectively preventing the colonization and reproduction of harmful bacteria [30,31]. By determining the amino acid distribution of TSP and TSPb, it was found that the hydrophobic amino acid contents of TSP and TSPb were categorized as 25.03 g and 30.04 g/100 g, with Ala and Pro being the most abundant, up to 7–10 g/100 g (Figure 2A).

To evaluate the effect of aminoacidic composition on antimicrobial activity, the IR spectra of TSP and TSPb were determined. Compared to TSP, TSPb showed redshifts in the amide I (near 1634 cm^−1^) and amide II (near 1530 cm^−1^) regions, and a blueshift in the amide III (near 1335 cm^−1^) region, with minimal changes in the absorbance intensity (Figure 2B). Similar to most antimicrobial peptides, both TSP and TSPb predominantly adopted α-helical and β-sheet secondary structures [30] (Figure 2C). TSPb exhibited four β-sheet absorption peaks (1609, 1618, 1629, and 1638 cm^−1^) and one α-helix absorption peak (1653 cm^−1^), with the α-helix red-shifted with respect to TSP, and the β-sheet at 1638 cm^−1^ was unique to TSPb (Figure 2C).

### 3.4. Impacts on H. pylori Membrane Integrity and Nucleic Acid Leakage

#### 3.4.1. Membrane Integrity

**Figure 3:**
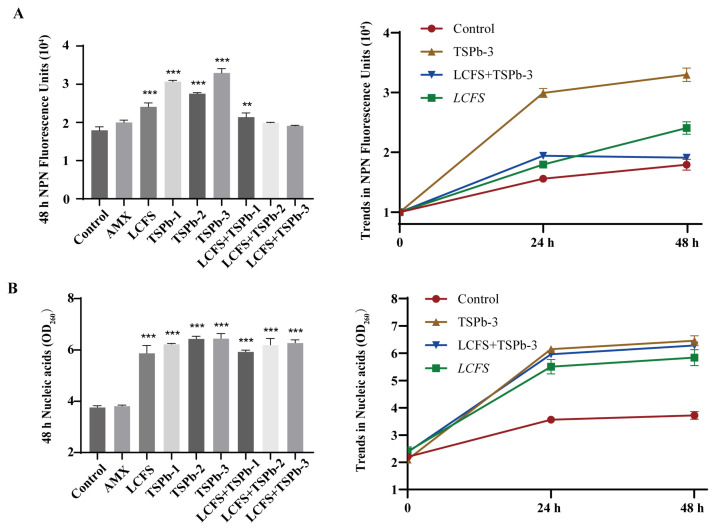
The effects of different treatments on the *H. pylori* cell membrane integrity and nucleic acid leakage. (**A**) NPN fluorescence units after 48 h and the trends of the changes (*n* = 3). (**B**) Nucleic acid leakage after 48 h and the trends of the changes (*n* = 3). ANOVA, ** *p* < 0.01, and *** *p* < 0.001 vs. the Control group.

The membrane integrity of *H. pylori* was assessed using the hydrophobic fluorescence probe NPN, which exhibits a positive correlation between the fluorescence intensity and NPN content [28]. The results showed that AMX had a relatively minor effect on the biofilm integrity of *H. pylori* compared to the control group. However, in the TSPb-treated group, the NPN content was significantly increased, with the highest levels observed in the TSPb-3 group (32,964.33 ± 1123.01), which were 1.84 times higher than those of the control group, indicating typical membrane-disruptive effects. In contrast, the LCFS group displayed a lower fluorescence intensity, suggesting that its mechanism of action might be non-membrane-disruptive. In the LCFS + TSPb group, the fluorescence intensity was reduced compared to the groups treated with LCFS or TSPb alone, indicating potential changes in the internal composition of the membrane (Figure 3A).

#### 3.4.2. Nucleic Acid Leakage

Bacterial death results from nucleic acid leakage from the cell [32]. Nucleic acid leakage from *H. pylori* cells due to the actions of LCFS and TSPb was analyzed by measuring the OD at 260 nm. As the incubation time increased, no significant trend of nucleic acid leakage was observed in the AMX group. Compared to the control group, *H. pylori* cells treated with TSPb, LCFS, and their combination exhibited significant nucleic acid leakage, which increased in a concentration-dependent manner. The degree of nucleic acid leakage in *H. pylori* cells was significantly enhanced when LCFS was combined with different concentrations of TSPb, suggesting that TSPb potentiates the effect of LCFS (Figure 3B). Meanwhile, the level of nucleic acid leakage showed an upward trend within 48 h, but the rate of increase slowed down after 24 h. Based on the results shown in Figure 3A,B, LCFS and TSPb have significantly different effects on bacterial membrane integrity, while exhibiting a comparable and significant impact on nucleic acid leakage. This indicates that their mechanisms of action are distinct.

### 3.5. Biofilm Morphology of H. pylori

**Figure 4:**
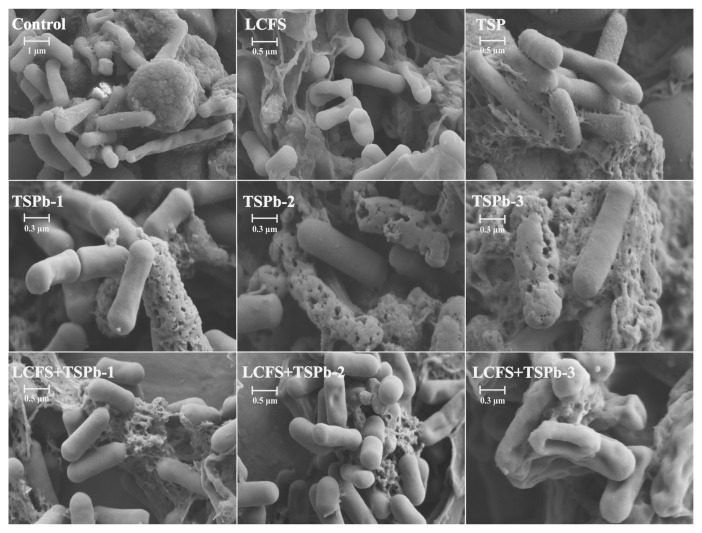
SEM morphology of H. pylori after 48 h of different treatments.

The morphological changes of *H. pylori* were assessed using scanning electron microscopy (Figure 4). In untreated *H. pylori*, the surface was smooth, elongated in shape, and the cell membrane was intact. After treatment with TSP, irregularities in the bacterial morphology and surface structure were observed. In the TSPb-treated group, distinct holes were evident on the bacterial membrane surface, with a severe disruption of membrane integrity. As the concentration of TSPb increased, the holes on the bacterial membrane enlarged, eventually leading to large fissures. This result also corroborated the conclusion from the NPN fluorescence assay in Section 3.4.1, where TSPb treatment led to compromised membrane integrity. After LCFS treatment, the bacterial surface exhibited varying degrees of indentation and deformation, showing a trend towards “softening”, while the membrane remained relatively intact, consistent with the conclusions in Section 3.4.1. Under the combined LCFS and TSPb treatment, *H. pylori* cells showed severe shrinkage and extensive inward membrane retraction, resembling a state of “dehydration”. Compared to the LCFS-only treatment, the combined treatment resulted in more pronounced membrane deformation.

### 3.6. Bacterial Composition Analysis

**Figure 5:**
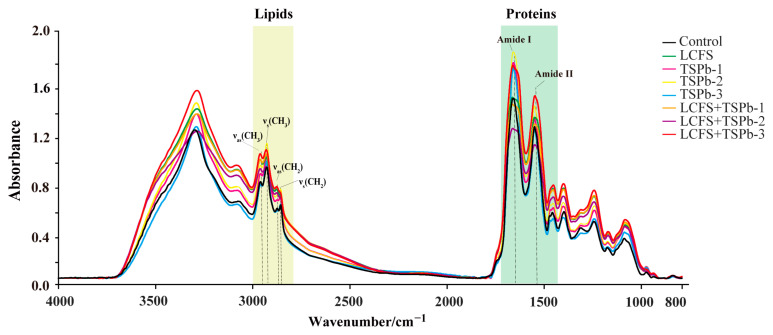
IR spectra of *H. pylori* from 4000 to 800 cm^−1^.

The changes in the chemical composition of the sample were encompassed by infrared transmission imaging, thereby reflecting alterations in its internal structure [33]. The IR spectral results for *H. pylori* after being treated with of TSPb and LCFS alone or in combination are shown in Figure 5. Significant differences were observed in both shape and position of the protein peaks (1700–1500 cm^−1^), while noticeable changes in peak intensities were observed in the lipid region (3000–2800 cm^−1^) (Table 2). To further investigate the structural changes in *H. pylori*, an additional infrared spectroscopy analysis was required.

#### 3.6.1. Proteins

**Figure 6:**
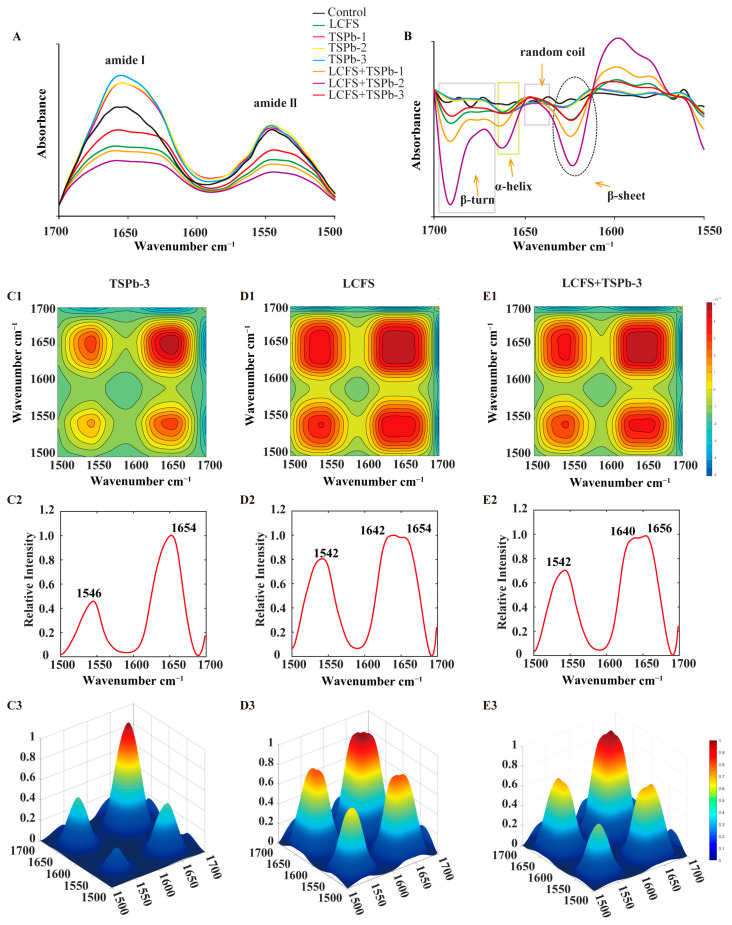
(**A**) IR spectra of *H. pylori* from 1700 to 1500 cm^−1^. (**B**) SD-IR spectra of *H. pylori* from 1700 to 1500 cm^−1^. (**C**–**E**) 2DCOS-IR spectra (1), 2D autopeaks (2), and 3D autopeaks (3) of *H. pylori* from 1700 to 1500 cm^−1^.

In the range of 1700–1500 cm^−1^, the absorption peaks primarily attributed to the amide I region (1700–1600 cm^−1^) and the amide II region (1600–1500 cm^−1^) were observed, corresponding to protein structures [34]. Treatment with TSPb increased the intensity of these two characteristic peaks and induced a redshift, while the addition of LCFS resulted in decreased peak intensities and broadening of the peaks. Notably, only the LCFS + TSPb-3 group exhibited an increase in protein absorption peaks compared to LCFS treatment alone (Figure 6A). Changes in the protein secondary structure predominantly occurred near the amide I band, which can be attributed to dipole coupling resulting from oscillations between carbonyl groups. *H. pylori* displayed absorptions in all four secondary structure regions, while TSPb and LCFS led to the disappearance of random coil absorption and the merging of two absorption peaks for β-turns (Figure 6B).

The 2DCOS-IR spectra exhibited changes in the spectral intensity resulting from variations induced by the addition of drugs. Notably, the intensity of these autopeaks became stronger as the changes in the *H. pylori* samples became more pronounced [35]. Through the analysis of the two-dimensional correlation spectra, we observed strong autopeaks near 1654 and 1546 cm^−1^ in all three groups. TSPb-3 facilitated the transition of random coil absorption in the LCFS group. Consequently, we inferred that under the influence of LCFS, the broad peak formed near the amide I region encompassed two peaks. Initially, LCFS induced a blue shift in the random coil absorption, resulting in a peak at 1642 cm^−1^. Subsequently, with the addition of TSPb-3, the blue shift in the random coil absorption intensified to 1640 cm^−1^, while the remaining portion shifted to 1656 cm^−1^. These shifts correspond to the transformation of random coil absorption into α-helices and β-sheets, respectively. Furthermore, the blue shift observed in the amide II region was primarily caused by LCFS (Figure 6C–E).

#### 3.6.2. Lipids

**Figure 7:**
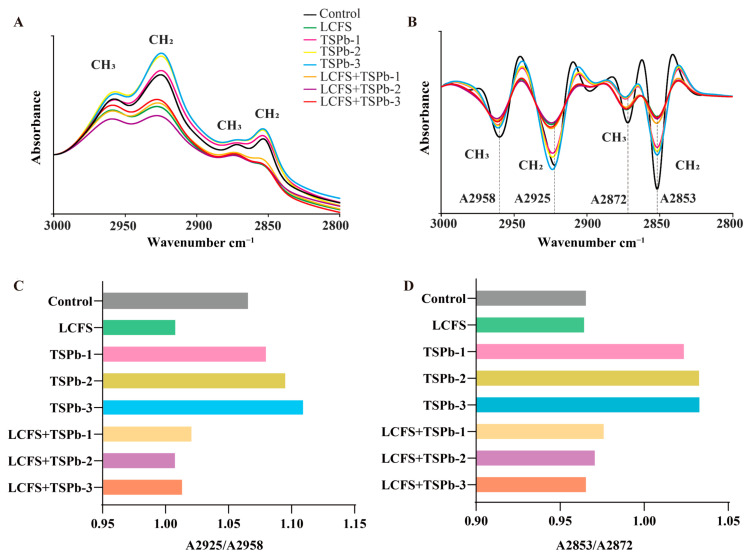
(**A**) IR spectra of *H. pylori* from 3000 to 2800 cm^−1^. (**B**) SD-IR spectra of *H. pylori* from 3000 to 2800 cm^−1^. (**C**) The ratio of the peak intensity of *H. pylori* at 2925 and 2958 cm^−1^. (**D**) The ratio of the peak intensity of *H. pylori* at 2853 and 2872 cm^−1^.

**Figure 8:**
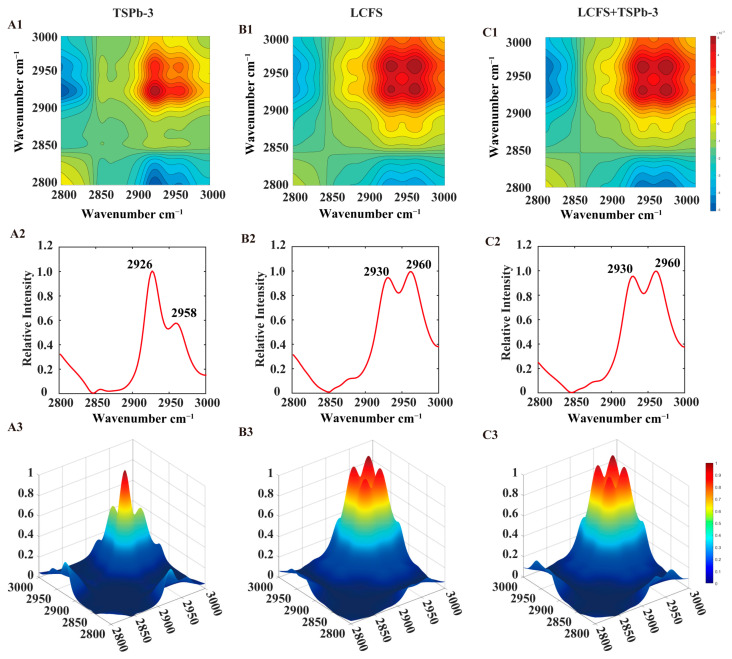
(**A**–**C**) 2DCOS-IR spectra (1), 2D autopeaks (2), and 3D autopeaks (3) of *H. pylori* from 3000 to 2800 cm^−1^.

The absorption region in the 3000–2800 cm^−1^ range, which mainly corresponded to lipid chains such as phospholipids and fatty acids, reflected the C-H stretching vibrations in organic matter [36]. The ratio of peak intensities between 2925 and 2958 cm^−1^ and between 2853 and 2872 cm^−1^ could be used to analyze the changes in the composition of lipids and biofilms in bacteria [37]. In contrast to the observed increase in the intensity of the four absorption peaks following TSPb treatment, both the LCFS and mixed group exhibited a decrease in peak intensity accompanied by peak broadening (Figure 7A). As shown in Figure 7B), it becomes apparent that the individual or combined treatments with TSPb and LCFS displayed similar characteristics, with A2958, A2925, and A2872 exhibiting a blue shift, while A2853 remained relatively unchanged. However, their peak ratios exhibited significant differences. TSPb induced a concentration-dependent increase in the peak ratios of A2925/A2958 and A2853/A2872, whereas the addition of LCFS resulted in a noticeable decrease in both peak ratios, with A2925/A2958 showing no concentration dependence (Figure 7C,D). The analysis of 2DCOS-IR spectra unveiled the crucial role of asymmetric stretching vibrations of C-H in lipid changes. The effects of TSPb-3 on *H. pylori* were primarily observed near 2925 cm^−1^, while LCFS exhibited effects near 2958 cm^−1^ (Figure 8A–C).

## 4. Discussion

*H. pylori* has been classified as a human carcinogen, posing a significant public health challenge due to its association with chronic gastritis, peptic ulcers, and gastric cancer. The effective prevention and treatment of *H. pylori* infections are therefore of critical importance. Clinical studies have demonstrated that AMPs and probiotics can enhance *H. pylori* eradication rates while minimizing antibiotic-associated side effects [17,38].

Currently, antimicrobial peptides extracted from milk casein are released in the stomach through enzymatic hydrolysis, demonstrating significant inhibitory effects against *H. pylori* [39]. Anti-adhesive peptides derived from rice bran protein exhibit potent anti-infective properties by binding to the adhesion factors of *H. pylori*, preventing bacterial adhesion to gastric cells and thereby inhibiting infection [40]. Food-derived antimicrobial peptides show great potential for development in the field of *H. pylori* control. Furthermore, previous studies have shown that *Lactobacillus plantarum WUH3* contains various bioactive antimicrobial substances (such as bacteriocins, lactic acid, hydrogen peroxide, etc.), and the bacteriocin Plantaricin EF has been successfully identified. Plantaricin EF exerts its action on bacterial cell membranes, leading to membrane polarization and modification [13]. It is a class II bacteriocin composed of two peptide subunits, which interacts electrostatically with negatively charged phospholipid molecules and inserts into the lipid bilayer. This interaction induces membrane structural loosening, reduces mechanical tension, increases fluidity, and significantly enhances membrane permeability.

In this study, we successfully isolated TSPb through enzymatic digestion and ultrafiltration, and further demonstrated its significant anti-*H. pylori* activity both individually and in combination with LCFS [41].

TSPb showed strong destructiveness to the survival environment of *H. pylori*, particularly by targeting urease activity, a critical virulence factor for bacterial colonization. Urease hydrolyzes urea to ammonia, neutralizing gastric acid, creating a favorable environment for *H. pylori* and damaging the gastric mucosa, which enhance bacterial virulence [42,43]. The significant disparity in the urease activity inhibition rate between the low-concentration TSPb group and the combination group suggested that the early disruption of *H. pylori*’s survival environment by LCFS may have enhanced the effectiveness of TSPb. This notion was further substantiated by the relatively slower growth rate of urease activity inhibition by LCFS compared to TSPb within the 24–48 h period.

The formation of antibiotic resistance is partly attributed to the limited permeability caused by the bacterial outer membrane. As a crucial component of the cell wall, the outer membrane protects the inner membrane and cytoplasmic membrane from external environmental stresses, thereby maintaining bacterial structural stability [14,44]. NPN, a hydrophobic fluorescent dye, exhibits significantly enhanced fluorescence in the hydrophobic environment of the bacterial outer membrane [45]. Interestingly, the NPN fluorescence intensity of the LCFS-treated group and the combination-treated group was lower than that of the control group. In the combined treatment group, the fluorescence intensity further decreased with the increase in the TSPb concentration. However, the nucleic acid leakage assay showed a significant increase in nucleic acid release in both the LCFS group and the combined treatment group, indicating that membrane permeability was significantly enhanced even without severe membrane disruption. Specifically, treatment with TSPb-3 alone resulted in localized membrane damage, exposing hydrophobic regions and leading to a marked increase in the NPN fluorescence intensity. In contrast, combined treatment with LCFS reduced the NPN fluorescence intensity by altering the physicochemical properties of the membrane, such as reducing hydrophobic regions, redistributing membrane lipids, or modifying surface charges. Despite the decrease in NPN fluorescence, LCFS-induced membrane loosening and remodeling significantly increased overall permeability, as evidenced by the substantial leakage of nucleic acids, further confirming membrane structural disruption. The SEM analysis (corrected the previous problem made by the research group) revealed distinct differences in the antibacterial mechanisms of TSPb and LCFS. When used alone, TSPb exhibited a mode of action similar to conventional cationic antimicrobial peptides, disrupting membrane integrity by forming localized pores on the bacterial surface. In contrast, LCFS primarily altered the surrounding alkaline environment of *H. pylori*, inducing membrane loosening and increased permeability. Under the combined treatment, LCFS acted first on the bacterial membrane, inducing structural loosening and enhanced permeability, which facilitated TSPb penetration into the bacterial cells. TSPb subsequently disrupted critical metabolic processes within the cells, ultimately leading to severe membrane collapse, intracellular leakage, and bacterial shrinkage. SEM images clearly demonstrated that *H. pylori* treated with the combination therapy exhibited extensive membrane rupture and severe cellular deformation. The synergistic mechanism involves LCFS first loosening the bacterial membrane, thereby facilitating TSPb penetration and subsequent disruption of intracellular metabolic processes. Unlike traditional antibiotics (e.g., β-lactams) that primarily target actively dividing bacteria, this combined approach is also effective against metabolically inactive or dormant bacteria. Therefore, this strategy demonstrates significant advantages in combating biofilm-associated and persistent bacterial infections, particularly in eradicating recalcitrant strains with remarkable efficacy.

*H. pylori*, as a representative Gram-negative bacterium, exhibited a substantial presence of phospholipids on the biofilm membrane. The structure and composition of the biofilm play significant roles in regulating permeation, growth, division, and other fundamental processes in *H. pylori* [46,47]. Monitoring changes in lipids and proteins in *H. pylori* through infrared spectroscopy provided valuable insights into its metabolic state [33]. We postulated that the abundant α-helices and β-sheets in TSPb-3 were pivotal in driving structural changes in *H. pylori* within the LCFS group. The action of TSPb-3 induced a blue shift in the characteristic lipid absorption peaks A2958, A2925, and A2872, accompanied by an increase in the ratios of A2925/A2958 and A2853/A2872. These alterations indicated an elevated proportion of methylene (CH2) relative to methyl (CH3), expansion of fatty hydrocarbon chains, increased unsaturation of fatty acids, and enhanced fluidity and permeability of the biofilm [36]. Studies have shown that antimicrobial peptides interact with the lipid bilayer through hydrophobic interactions. When the lipids contain unsaturated fatty acids, the fluidity of the membrane is enhanced, which may lead to the formation of pores on the membrane surface, thereby increasing membrane permeability [19]. Although LCFS also induced a blue shift of A2958, A2925, and A2872, the A2925/A2958 and A2853/A2872 ratios were reduced. Based on the IR spectroscopy results, LCFS treatment may have induced a modification of the surface structure of the bacterial membranes, leading to the relaxation and softening of the membranes rather than localized membrane rupture. This loosening and increased porosity of the membrane led to the partial release of lipids into the supernatant, causing changes in bacterial morphology, characterized by shorter and rounder cells. Consequently, the overall cell volume and relative surface area decreased, ultimately leading to a reduction in the characteristic peak ratios.

In summary, this study revealed the unique and synergistic antibacterial mechanisms of TSPb and LCFS against *H. pylori*, highlighting their complementary roles in disrupting the bacterial membrane structure, enhancing membrane permeability, and interfering with bacterial metabolic processes. TSPb exerted a direct disruptive effect by forming localized pores on the membrane surface, while LCFS induced membrane loosening and increased porosity, facilitating the efficient penetration of TSPb into bacterial cells and significantly enhancing the bactericidal efficacy. This combined treatment not only accelerated bacterial death but also exhibited notable advantages against metabolically inactive or biofilm-embedded bacteria, overcoming the limitations of conventional antibiotics. The findings of this study provide a solid scientific basis for the application of natural antimicrobial agents in the field of gastric health. Furthermore, they demonstrate the potential of innovative dietary supplement strategies in the prevention and management of *H. pylori* infections. By integrating multiple natural bioactive components, this approach advances the development of functional foods aimed at improving gastrointestinal health and offers a sustainable, food-based intervention pathway for enhancing human health.

## 5. Conclusions

The results of this study demonstrated that TSPb, a highly bioactive peptide fraction extracted from *Takifugu obscurus* skin, and the cell-free supernatant (LCFS) of *Lactobacillus plantarum WUH3* exhibit significant anti-*H. pylori* activity. Both treatments effectively reduced urease activity, disrupted the bacterial biofilm, and altered the membrane structure. Specifically, the combined treatment achieved a urease inhibition rate of 74.65% at 24 h and 78.42% at 48 h, which were significantly higher than the individual treatments. The SEM analysis revealed that LCFS induced structural loosening and deformation of the bacterial membrane surface, while TSPb caused localized pore formation, resulting in mild membrane damage. IR spectroscopy further confirmed the significant disruption of lipid and protein structures, causing severe membrane modification and increased permeability, which ultimately resulted in bacterial death.

These results highlight the potential of TSPb and LCFS as safe, natural, and bioactive dietary supplements for the prevention and management of *H. pylori* infections. By leveraging their complementary mechanisms of action, this combination offers a sustainable and antibiotic-free strategy to address *H. pylori*-associated gastric disorders. This study provides a solid foundation for the development of functional foods designed to promote gastric health, offering a promising solution for improving gastrointestinal well-being through innovative and effective approaches.

## Figures and Tables

**Table 1:** Inhibition of *H. pylori* by TSP (*n* = 4).

Group	Diameter of Inhibition Zone (cm)
AMX (0.125 mg/mL)	1.76 ± 0.25
TSP-1 (1.000 mg/mL)	0.50 ± 0.10
TSP-2 (2.000 mg/mL)	0.70 ± 0.10
TSP-3 (3.000 mg/mL)	0.94 ± 0.05

**Table 2:** Distribution of the main characteristic absorption peaks of the IR spectrum of *H. pylori* under different treatment conditions.

Peak Position (cm^−1^)	Main Functional Groups
~3289	*υ* (NH_3_)
3000–2800	*υ_as_* (CH_3_), *υ_as_* (CH_2_);*υ_s_* (CH_3_), *υ_s_* (CH_2_)
1700~1600	Amide I
1600~1500	Amide II
~1460	*υ_as_* (CH_3_), *υ* (CH_2_)
~1395	*δ_s_* (CH_3_), *υ_s_* (COO-)
1085, 1240	*υ_s_* (PO_2_^−^), *υ_as_* (PO_2_^−^)
1085–1041	*δ* (C-C), *υ* (C-O)
980–930	*δ_out-plane_* (C-H), *δ* (C-O), *υ* (C-C)

*υ*: stretching vibration, *δ*: flexural vibration, *s*: symmetric, *as*: antisymmetric.

## Data Availability

The original contributions presented in the study are included in the article further inquiries can be directed to the corresponding author.
